# Evaluating bias due to data linkage error in electronic healthcare records

**DOI:** 10.1186/1471-2288-14-36

**Published:** 2014-03-05

**Authors:** Katie Harron, Angie Wade, Ruth Gilbert, Berit Muller-Pebody, Harvey Goldstein

**Affiliations:** 1 Institute of Child Health, University College London, 30 Guilford Street, London WC1 N 1EH, UK; 2 Public Health England, London, UK; 3 Institute of Child Health, University College London and University of Bristol, Bristol, UK

**Keywords:** Data linkage, Routine data, Bias, Electronic health records, Evaluation, Linkage quality

## Abstract

**Background:**

Linkage of electronic healthcare records is becoming increasingly important for research purposes. However, linkage error due to mis-recorded or missing identifiers can lead to biased results. We evaluated the impact of linkage error on estimated infection rates using two different methods for classifying links: highest-weight (HW) classification using probabilistic match weights and prior-informed imputation (PII) using match probabilities.

**Methods:**

A gold-standard dataset was created through deterministic linkage of unique identifiers in admission data from two hospitals and infection data recorded at the hospital laboratories (original data). Unique identifiers were then removed and data were re-linked by date of birth, sex and Soundex using two classification methods: i) HW classification - accepting the candidate record with the highest weight exceeding a threshold and ii) PII–imputing values from a match probability distribution. To evaluate methods for linking data with different error rates, non-random error and different match rates, we generated simulation data. Each set of simulated files was linked using both classification methods. Infection rates in the linked data were compared with those in the gold-standard data.

**Results:**

In the original gold-standard data, 1496/20924 admissions linked to an infection. In the linked original data, PII provided least biased results: 1481 and 1457 infections (upper/lower thresholds) compared with 1316 and 1287 (HW upper/lower thresholds). In the simulated data, substantial bias (up to 112%) was introduced when linkage error varied by hospital. Bias was also greater when the match rate was low or the identifier error rate was high and in these cases, PII performed better than HW classification at reducing bias due to false-matches.

**Conclusions:**

This study highlights the importance of evaluating the potential impact of linkage error on results. PII can help incorporate linkage uncertainty into analysis and reduce bias due to linkage error, without requiring identifiers.

## Background

Linkage of records between electronic health databases is becoming increasingly important for research purposes as individual-level electronic information can be combined relatively quickly and inexpensively [1,2]. The success of such data linkage depends on data quality, linkage methods, and the ultimate purpose of the linked data [3]. Errors that occur during the linkage process (false-matches and missed-matches) can lead to biased results, although the extent of this bias in research based on linked data is difficult to measure, as reported measures of linkage error (e.g. sensitivity, specificity, match rate) do not necessarily allow us to understand the impact of these linkage errors on results [4-8]. The separation of linkage and analysis (to protect data confidentiality) means that researchers analysing linked datasets often lack the information required to properly assess the impact of error on results [9,10].

When data do not include well completed or accurate unique identifiers, probabilistic match weights are often used to measure the similarity between records in different files [11,12]. Match weights represent the likelihood of records being a match given the agreement of a set of identifiers. Typically, records are then classified as links or non-links by retaining the candidate with the highest weight, given the weight exceeds a specified cut-off threshold (highest-weight classification). The choice of thresholds directly affects the number of false-matches and missed-matches in linked data.

Several alternatives to highest-weight classification that aim to adjust for linkage bias have been proposed, but these are generally limited to the context of regression analysis [13-16]. A more flexible method for dealing with linkage uncertainty when analysing linked data is prior-informed imputation (PII) [17]. PII aims to select the correct value for variables of interest, and rather than accepting a single complete record as a link, allows more than one candidate linking record to be considered in analysis. Information from match probabilities in candidate linking records (the prior) is combined with information in unequivocally linked records. This process avoids errors associated with accepting the wrong record as a link, or failing to accept any record as a link. PII has been shown to work well in a simulation study involving linear regression but has not yet been evaluated using real data or explored in different linkage and analysis situations.

Determining the potential effect of linkage error on relevant outcome measures is vital if linked data are to be used in health research. We evaluate the impact of linkage error on analysis of infection rates in paediatric intensive care, based on a national audit dataset (PICANet, the Paediatric Intensive Care Audit Network) and infection surveillance data linked using highest-weight (HW) classification and prior-informed imputation (PII) [18]. Simulated data are used to investigate how the impact of linkage error varies according to the characteristics of the data to be linked.

## Methods

### Ethics

For PICANet, collection of personally identifiable data has been approved by the Patient Information Advisory Group (now the NHS Health Research Authority Confidentiality Advisory Group) http://www.hra.nhs.uk/documents/2013/11/piag-register-2.xls and ethical approval granted by the Trent Medical Research Ethics Committee, ref. 05/MRE04/17 +5. PICANet also has specific permission from the National Research Ethics Service for linkage with the PHE laboratory data on bloodstream infections using personal identifiers and to share PICANet data with PHE. An exemption under Section 251 of the NHS Act 2006 (previously Section 60 of the Health and Social Care Act 2001) allows PHE to receive patient-identifiable data from other organisations without patient consent in order to monitor infectious disease. Specific permission for the PICANet-PHE linkage has been granted by NIGB. Consent for the use of the data identifying individual PICUs in this study was obtained from the PICU leads.

### Statistical analysis

The primary outcome was PICU-acquired blood-stream infection (BSI), defined as any positive blood culture occurring between 2 days after PICU admission and up to 2 days following PICU discharge inclusive. The crude rate of PICU-acquired BSI was calculated as the number of events per 1000 bed-days (only one event counted per admission). Poisson regression models were fitted to the data to estimate the absolute difference in adjusted rates between hospitals. Variables known to be associated with PICU-acquired BSI in these datasets were included in models. Statistical analysis was performed using Stata 11 [19].

### Original data

Admission data for children admitted to Birmingham Children’s hospital (BCH) or Great Ormond Street hospital (GOSH) paediatric intensive care units (PICUs) between March 2003 and December 2010 were extracted from the PICANet database [18]. Microbiology records for all positive bacterial isolates from blood were obtained from BCH and GOSH laboratories for March 2003 to December 2010. Deterministic linkage of PICANet and microbiology records based on unique identifiers (National Health Service (NHS) number, hospital number, name, date of birth and sex) provided the true match status of each record pair. The original “gold-standard” dataset consisted of every PICANet record and linked microbiology records where a link existed. Linkage was manually verified to ensure there were no false-matches or missed-matches and additional data from the hospital IT systems (e.g. examination of previous names) were used to clarify any uncertainties.

### Simulated data

**Figure 1:**
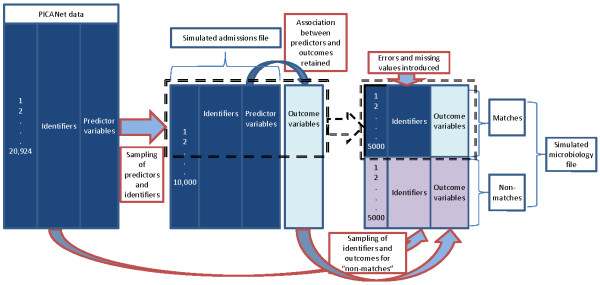
Creation of simulated data.

To evaluate methods for linking data with different characteristics, we generated a second “admissions” file of 10,000 records by randomly sampling 10,000 values for each of the identifiers in the original PICANet data (Figure 1). Several sets of twenty-five simulated “microbiology” files were then created by sampling 10,000 records from PICANet, and selecting a set number of these records to have a link in the admissions file – i.e. representing positive blood cultures occurring within a PICU admission. Each set of simulated microbiology files was given different attributes to reflect the range of linkage situations and data quality that might be expected of linkage between routine data sources (Table 1). The match rate was set to either 10%, 50% or 70% by selecting 1000, 5000 or 7000 records in the microbiology file to link to an admission record. Identifier values were randomly changed (completely different values entered) and missing values randomly introduced into either 5% or 10% of records. Finally, the distribution of error was set to be either random or non-random. Non-random error was introduced by hospital (microbiology records from hospital 1 were 5 times more likely to include error than records from hospital 2) or non-random by outcome (microbiology records linking to a PICU admission were 5 times more likely to have error than those not linking to a PICU admission).

**Table 1:** Description of original and simulated datasets

**Dataset**	**Error distribution**	**Match rate**	**Error rate**
**Original data (PICANet-LabBase2)**	Error varied by hospital	Matches: 1496/20924 (7%)	0-5% error,
		Non-matches: 19431/20924 (93%)	<1% missing values
**Simulated datasets**			
1	Random identifier error		
2	Non-random error (associated with hospital)	Matches: 1000/10000 (10%)	5% error,
	Non-random error (associated with outcome)	Non-matches: 9000/10000 (90%)	5% missing values
3			
4	Random identifier error		
5	Non-random error (associated with hospital)	Matches: 5000/10000 (50%)	5% error,
	Non-random error (associated with outcome)	Non-matches: 5000/10000 (50%)	5% missing values
6			
7	Random identifier error		
8	Non-random error (associated with hospital)	Matches: 7000/10000 (70%)	5% error,
9	Non-random error (associated with outcome)	Non-matches: 3000/10000 (30%)	5% missing values
10	Random identifier error		
11	Non-random error (associated with hospital)	Matches: 1000/10000 (10%)	10% error,
12	Non-random error (associated with outcome)	Non-matches: 9000/10000 (90%)	10% missing values

All data were linked using both highest-weight classification and PII.

### Linkage

Although BCH and GOSH laboratories were able to provide data with well-completed and discriminatory identifiers, national infection surveillance data includes more limited information, as NHS number, hospital number, name and postcode are often not recorded. To simulate the linkage approach required for the national surveillance data, we removed unique identifiers from all files. Linkage was then based on sex, Soundex (an anonymised phonetic code for surname [20]) and day, month and year of birth only.

In each case, the admissions file consisted of a cohort of children admitted to PICU. The linking microbiology file held records for children who had one or more positive blood cultures at one of the two hospitals, some of whom had spent time in PICU. Admission records could therefore link to none, one or more records in the microbiology file. The variable of interest (VOI) was ‘BSI’, coded in the gold-standard data as 1 for admissions having a link in the microbiology file, and 0 for admissions not having a link in the microbiology file.

### Classification method 1: highest-weighted

Probabilistic match weights were assigned to each record pair, according to agreement on the set of identifiers (Fellegi-Sunter approach) [21]. Match weights were calculated using the conditional probability that a record pair agree on a particular identifier, given the true match status of the pair e.g. P(agree on sex|match) and P(agree on sex|non-match). Since in our gold-standard data the true-match status of each record pair was known, conditional probabilities were calculated directly. Match weights were calculated using code written in Stata 11 [19].

Record pairs were ordered by descending weight and the highest-weighted pair for each admission and microbiology record was accepted as a link, provided the weight exceeded a specified cut-off threshold. Where an admission record linked to more than one microbiology record with equal weight, the earliest microbiology record was retained (only one event per admission was counted). Where a microbiology record genuinely linked to more than one admission record (e.g. for consecutive admissions), the earliest admission was retained. Record pairs with weights below the threshold were classified as non-links.

Thresholds are typically chosen by ordering record pairs by weight and manually inspecting to determine the weight at which pairs were thought to be more likely than not a match. Two thresholds are chosen, and record pairs with weights between the two thresholds are subjected to manual review. However in national infection surveillance data, manual review is not feasible–firstly due to the large numbers of records, and secondly due to the scarcity of identifying information on records (decisions would need to be based on few identifiers, e.g. only Soundex and date of birth).

If linkage error rates are known, a single threshold can be chosen to minimise linkage error. An optimal threshold would either minimise the sum of errors (false-matches + missed-matches) or minimise the net effect of errors (|false-matches–missed matches|). Unfortunately, it is not always possible to obtain estimates of linkage error rates with which to derive optimal thresholds. However, if error rates are available in a subset of data (e.g. from a gold-standard dataset), these can be used to select a threshold.

For each linkage, a 10% subset of records where the true match status was known was used to estimate the number of false-matches and missed-matches at each possible weight threshold. Optimal thresholds based on this subset were then applied to the entire dataset. In the simulated data, the threshold that minimised the sum of errors (threshold 1) and the threshold that minimised the net effect of errors (threshold 2) coincided, and results are presented from one threshold only.

### Classification method 2: prior-informed imputation

PII was performed as proposed by Goldstein et al., using Stat-JR software developed by the University of Bristol [22]. A detailed description of the method has been published elsewhere and further details relating to this study are provided as an Additional file 1[17]. PII works by transferring values of variable(s) of interest (VOI) from the linking file to a primary analysis file, rather than linking to a complete record. In this analysis, the VOI was a binary variable corresponding to infection, recorded at either GOSH or BCH. If an admission record linked to a microbiology record there was assumed to be an infection and the VOI was coded as 1. If there was no link, there was no infection and the VOI was coded as 0.

Match probabilities were derived from the probability that records were a match given the joint agreement of a set of identifiers e.g. P(M|agree on sex and Soundex and date of birth), based on the true match status of record pairs. This joint estimation avoids the assumption of independence between identifiers that can result in misclassification of record pairs [23].

For admission records that had an unequivocal link in the microbiology file (match probability > 0.9), the VOI value associated with the linking record was accepted. In this analysis, the VOI was set to 1 as any record with a link in the microbiology file represented an admission with an infection. For admission records that were unequivocally non-links (match probability < 0.2), the VOI was set to 0, as there was no infection. These cut-off probabilities were based on previous PII simulation work.

For admission records that had more than one candidate linking record (equivocal links), a prior distribution for the VOI was derived from the match probabilities associated with each candidate record. In this ‘incomplete linkage’, any record that had a match in the microbiology file had a BSI. Therefore the value of the VOI was the same for all candidate records (i.e. the VOI = 1). The maximum candidate probability reflects the maximum probability of BSI for an individual record, and so the prior was derived as:

$$\mathrm{VOI}=\left\{{}_{0,\;\mathrm{with}\;1‒P\left(\max\left[\mathrm{candidate}\;\mathrm{probabilities}\right]\right)}^{1,\;\mathrm{with}\;P\left(\max\left[\mathrm{candidate}\;\mathrm{probabilities}\right]\right)}\right)$$

A likelihood for the VOI was derived from the distribution of the VOI in unequivocally linked records, conditional on predictor variables in the admissions file. Predictor variables included were those known to be associated with PICU-acquired BSI in these data (renal status, quarter-year at admission, age, admission type and admission source) [24].

**Figure 2:**
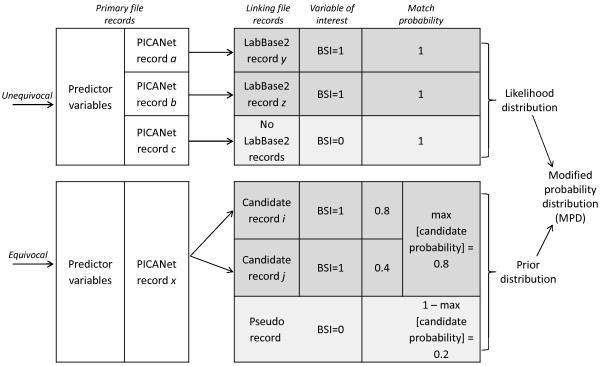
Prior-informed imputation for linkage of PICU and infection records.

A modified probability distribution (MPD) was then created, proportional to the prior distribution multiplied by the likelihood (Figure 2). For each equivocal admission record, the VOI associated with the highest value of the MPD was chosen. If no VOI value exceeded a pre-specified MPD threshold, the VOI was treated as missing and standard multiple imputation was used to impute a value based on the likelihood (unequivocal links) only.

The MPD threshold takes a standardised value from 0 to 1 and if no value exceeds the threshold, standard imputation is used to impute a value. If a low threshold is chosen (e.g. 0.1) the value of the VOI is almost always accepted from the MPD. If a high threshold is chosen (e.g. 0.9) the value of the VOI is almost always selected through standard multiple imputation (imputed from the likelihood). The choice of MPD threshold therefore determines how much precedence is given to information from the prior or the likelihood. Results from two MPD thresholds (0.1 and 0.9) are presented, to demonstrate this point. For each linkage, five imputed datasets were produced and analysed separately. Results were combined using Rubin’s rules [25].

## Results

### Original data

**Figure 3:**
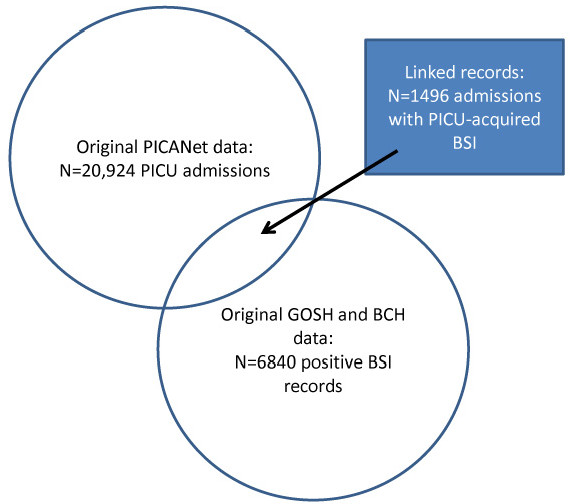
Linkage between PICANet and gold-standard microbiology data.

Of the 20924 admission records from March 2003 to December 2010 extracted from PICANet, 1496 (7.1%) linked to at least one microbiology record of PICU-acquired BSI (gold-standard data; Figure 3). Given a total of 116,113 bed-days, the rate of PICU-acquired BSI was 12.88 (95% CI 12.23-13.53) per 1000 bed-days; 11.18 (11.93-10.41) and 15.73 (16.87-14.55) at respective PICUs.

**Figure 4:**
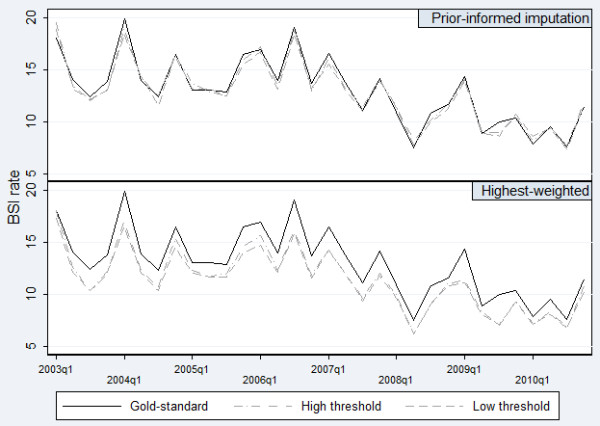
Comparison of crude PICU-acquired BSI rate obtained through highest-weighted classification and prior-informed imputation: original data.

After removal of unique identifiers, the number of PICU-acquired BSI was identified as 1316 (6.3%; HW threshold 1), 1287 (6.2%; HW threshold 2), 1481 (7.1%; PII 0.1) and 1457 (7.0%; PII 0.9). The crude rate of PICU-acquired BSI was identified as 11.33 (95% CI 10.72-11.95), 11.08 (10.48-11.69), 12.75 (11.61-13.89) and 12.55 (11.42-13.68) for HW threshold 1, HW threshold 2, PII 0.1 and PII 0.9 respectively. Incidence rates were underestimated when using HW (Figure 4).

The difference in adjusted rates between PICUs was 4.53 (95% CI 3.12-5.93) BSI per 1000 bed-days (gold-standard data). The difference in rates using HW threshold 1, HW threshold 2, PII (0.1) and PII (0.9) was 3.14 (1.84-4.45), 3.25 (1.95-4.55), 4.31 (2.62-6.00) and 4.18 (2.53-5.83) respectively. PII (0.1) provided the least biased results for these data.

### Simulated data

**Figure 5:**
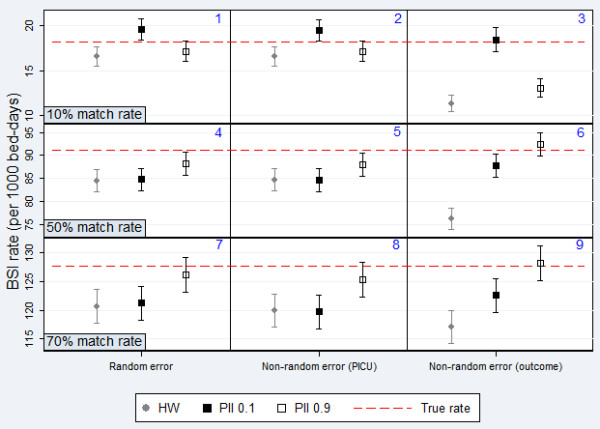
**Comparison of HW classification and PII for estimating BSI rate.** Data from simulated datasets 1-9; Symbols = point estimate; Lines = 95% confidence intervals. One extreme value for HW relaxed excluded (=49.08).

**Figure 6:**
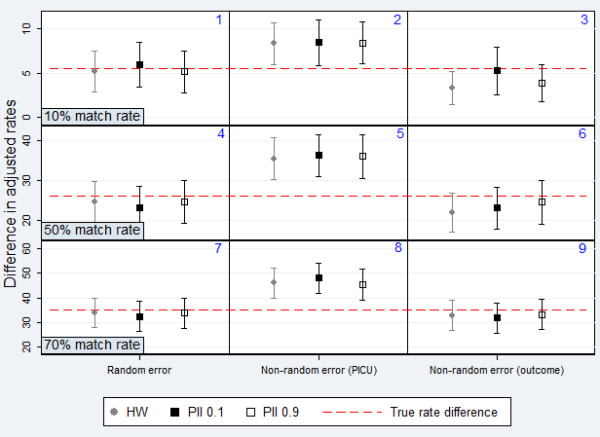
**Comparison of HW classification and PII for estimating the difference in adjusted rates between PICUs.** Data from simulated datasets 1-9; Symbols = point estimate; Lines = 95% confidence intervals.

Overall, results were most severely affected by linkage error when these errors were distributed non-randomly. Estimates of BSI rate were most biased when error was associated with the outcome of infection and HW classification was used (Figure 5). Estimates of the difference in adjusted rates were not significantly affected by random error, as errors were introduced to data from both PICUs equally (Figure 6). However substantial bias was introduced when error was associated with a particular hospital, as errors in data from one hospital led to an apparent lower rate and therefore falsely inflated the difference between units.

The size of the bias introduced to results also depended on the match rate. Estimates of BSI rate were biased by up to 8% for a match rate of 70%, rising to 38% for a match rate of 10% (Figure 5). Similarly, estimates of difference in rates were biased by up to 38% for a match rate of 70%, rising to 53% for a match rate of 10% (Figure 6). This difference was due to the fact that for 10% match rate, there were a greater number of non-matches and therefore more potential for false-matches.

For 10% match rate, PII (0.9) performed best, except for when error was associated with the outcome, where PII (0.1) provided least biased results. For 50% and 70% match rate respectively, PII (0.9) provided the least biased results in all cases but one, where HW performed marginally better.

Using PII rather than HW classification had most benefit when the proportion of true matches was lower, as in the original data. PII also performed well when the identifier error rate was increased to 10% (Table 2). In this case, the set of unequivocal links was less reliable and so the MPD threshold of 0.1 performed best as more weight was given to values in the candidate records. Standard errors for PII were generally larger than those for HW. This was due to the process of combining results from five multiply imputed datasets, and better reflects the uncertainty associated with linkage.

**Table 2:** Comparison of classification methods for estimating BSI rate and difference in adjusted rates with 10% identifier error (simulated datasets 10-12)

**Classification**	**N Infections**	**Crude rate (95% CI)** $\frac{N\times 1000}{54,826\;\mathrm{bed}\;\mathrm{days}}$	**Standard error**	**% bias**	**Difference in adjusted rates (95% CI)**	**Standard error**	**% bias**
*Gold standard*	*1000*	*18.24*	*0.577*		*5.514*	*1.214*	
**10: Random error**							
HW	869	15.84 (14.79, 16.90)	0.646	-13.1	4.55 (2.33, 6.76)	1.129	-17.5
PII MPD = 0.1	1038	18.94 (17.67, 20.21)	0.646	3.8	5.32 (2.75, 7.89)	1.313	-3.5
PII MPD = 0.9	860	15.69 (14.61, 16.77)	0.551	-14.0	4.45 (2.18, 6.72)	1.160	-19.3
**11: Non-random error; by covariate**							
HW	886	16.15 (15.09, 17.21)	0.543	-11.4	10.93 (8.61, 13.24)	1.183	98.2
PII MPD = 0.1	1010	18.41 (17.21, 19.62)	0.614	1.0	11.69 (9.12, 14.26)	1.311	111.9
PII MPD = 0.9	858	15.65 (14.57, 16.72)	0.548	-14.2	11.454 (9.09, 13.82)	1.208	107.7
**12: Non-random error; by outcome**							
HW	364	6.65 (5.98, 7.32)	0.343	-63.6	1.94 (0.53, 3.35)	0.720	-64.9
PII MPD = 0.1	684	12.48 (10.87, 14.09)	0.822	-31.6	3.36 (0.51, 6.20)	1.453	-39.1
PII MPD = 0.9	217	3.95 (3.39, 4.51)	0.287	-78.3	1.20 (0.00, 2.39)	0.610	-78.3

## Discussion

This study demonstrates that when linkage error due to missing or wrongly recorded identifiers is associated with a particular group of records, estimates based on linked data can be substantially biased. Considerable bias was also introduced when the match rate was low or when the identifier error rates were high. We show that in these cases, PII using match probabilities was able to produce less biased results compared with the traditional highest-weighted classification using probabilistic match weights.

In this study we assumed that both match weights and match probabilities were calculated accurately (i.e. based on the true match status of record pairs). In a real linkage situation this would not be the case, and the comparisons presented here therefore represent a best-case scenario. Further work needs to be done to understand how sensitive PII is to inaccuracies in match probabilities. Appropriate methods for estimating match probabilities and avoiding the assumption of independence between identifiers are currently being developed.

The benefit of using PII was most obvious in the results of linkage between the original data used in this study (PICANet and LabBase2). In the simulated data, PII with the high MPD threshold (0.9) generally provided the least biased results. Using a high MPD threshold means that VOI values are most often imputed from the likelihood, indicating that in some situations, standard multiple imputation would be sufficient for linkage. We recommend that the most effective classification method is chosen on a study-to-study basis, according to the characteristics of the data. This choice could also be informed by comparing results from each method with results from a subset of gold-standard data where the true match status of records is known, or by assessing in synthetic data with similar characteristics.

In many linkage situations, an individual may be recorded in each dataset to be linked, regardless of the outcome being studied (e.g. when GP records are linked to hospital records). PII has previously been shown to be effective at avoiding bias due to linkage error in such a situation [17]. In this study, records were only linked when the outcome was observed i.e. when a child admitted to PICU had BSI. Consequently, linkage error had a direct effect on the results calculated. In particular, bias was greatest when identifier error rates differed between hospitals, supporting other studies that have shown differential linkage error by ethnic group, exclusion of vulnerable populations due to poorly-recorded identifiers and erroneous rankings of relative hospital performance due to differing data quality between units [26-32]. These potential sources of bias need to be acknowledged to ensure transparent research based on linked electronic health data.

For linkage to be successful, communication between data linkers and users of linked data is vital. The separation principle, which means that data custodians are not allowed to release identifiable data to researchers and that linkage is performed by a third party, is advocated as good practice for confidentiality but means that researchers often lack the information needed to assess the impact of linkage error on results [9,33]. Data linkers need to be explicit about linkage methods, criteria, and any uncertainty in linkage. Linked data users need to consider what information is required to properly assess linkage bias.

Our evaluation of PII demonstrates that it is possible to handle linkage error without requiring access to any identifiable data, by retaining all candidate linking records and their associated match weights or probabilities [34]. Retaining match weights and candidate records would also allow sensitivity analyses using a range of linkage criteria (e.g. different thresholds in probabilistic linkage) to determine the effect of these criteria on results. Gold-standard datasets where true match status is known can be used to identify the most appropriate method for a linkage study, and to estimate measures of bias resulting from linkage error. Finally, access to both linked and unlinked records would allow the comparison of record characteristics to allow identification of potential sources of bias arising from associations between data quality and variables of interest [35].

## Conclusions

Linkage of routine data is a valuable resource for health research, but our study highlights the importance of evaluating the potential impact of linkage error on results. We show that PII can be used to help incorporate linkage uncertainty into analysis and to reduce bias due to linkage error, without requiring the release of individual identifiers. Improved methods for linkage and guidelines for evaluating and handling linkage error will help improve the reliability and validity of results based on linked data [36,37].

## Abbreviations

BCH: Irmingham Children’s hospital; BSI: Blood-stream infection; GOSH: Great Ormond Street hospital; HW: Highest-weighted; MPD: Modified probability distribution; PICANet: The Paediatric Intensive Care Audit Network; PICU: Paediatric intensive care unit; PII: Prior-informed imputation; VOI: Variable of interest.

## Competing interests

The author(s) declare that they have no competing interests.

## Authors’ contributions

KH carried out the analysis and wrote the first draft of the article. HG conceived of the study. AW, RG and BMP contributed to the study design and interpretation of the data. All authors critically revised the manuscript and approved the final version.

## Pre-publication history

The pre-publication history for this paper can be accessed here:

http://www.biomedcentral.com/1471-2288/14/36/prepub

## Supplementary Material

Additional file 1 Prior-informed imputation. Click here for file
