# Supplementary material for: Evaluating bias due to data linkage error in electronic healthcare records
Source: BMC Med Res Methodol. 2014 Mar 5;14:36. doi: 10.1186/1471-2288-14-36 (PMC4015706; doi:10.1186/1471-2288-14-36)
Supplement: Additional file 1 — Prior-informed imputation. [file 1471-2288-14-36-S1.docx]

**Supplementary file: Prior-informed imputation**

‘Prior-informed imputation’ (PII) is an extension of a method developed by Goldstein et al for combining a probability distribution with known data values in a multiple imputation framework [[1](#_ENREF_1)]. PII can be used for data linkage by allowing match weights or match probabilities to be incorporated into analyses of linked data.

In data linkage, records from different files are linked, in order to combine information relating to the same individual. Once a link is made, variable(s) of interest are transferred from the linking file(s) to the primary analysis file. PII works by ensuring that the probability distribution of the variable of interest (VOI) is transferred across to the primary file, rather than linking to a single value for a single candidate linking record.

In our study, each record in the PICANet file has a set of candidate linking records in the microbiology file, each with an associated match probability and associated value for our VOI (infection). Where a candidate linking record matched exactly on all identifiers, we called this an unequivocal link, and accepted the value of the VOI (i.e. infection). In highest-weighted classification, the record with the highest weight is accepted as a link and the value of the VOI associated with this record is transferred to the primary file. By contrast, PII uses information on the VOI values from all candidate linking records.

The set of candidate linking records for each PICANet admission record was created by selecting all microbiology records with an associated match probability >0.2. For PICANet records with more than one candidate linking record, the maximum candidate probability *p* was defined as the maximum of the match probabilities in all candidate linking records. Since some admission records did not have a genuine match in the laboratory file (not all admissions had an infection) the probability of not having a genuine match in the admissions file was also included in the probability distribution by creating a pseudo record with an associated linkage probability (Figure S1). This probability was calculated as 1-*p.*

For equivocal PICANet records, i.e. those with more than one candidate linking record, the true value of the VOI can be thought of as missing, but with an associated probability distribution that is a direct function of the set of match probabilities. This probability distribution forms a prior distribution for the VOI [[1](#_ENREF_1)]. This prior distribution is then combined with the (conditional) likelihood for the VOI based on the unequivocally-linked records.

For each equivocal record in the PICANet file, an MCMC algorithm is used to sample a value from the posterior distribution of the VOI, so that after every cycle of the algorithm we have a complete data set [[1](#_ENREF_1)]. A number of complete datasets (e.g. 10) are generated, and analysis is performed on each one. Estimates of the quantity of interest are combined using Rubin’s rules [[2](#_ENREF_2)].

|  |  | Candidate microbiology record 1 | Match weight 1 | Infection |
| --- | --- | --- | --- | --- |
| PICANet record *i* |  | Candidate microbiology record 2 | Match weight 2 | Infection |
|  |  | **.**  **.**  **.** | **.**  **.**  **.** | **.**  **.**  **.** |
|  |  | Pseudo microbiology record | Match weight 1-*p* | No infection |

Figure 1: Candidate linking records

**References**

1. Goldstein, H., et al., *Multilevel models with multivariate mixed response types.* Statistical Modelling, 2009. **9**(3): p. 173-197.

2. Rubin, D., *Multiple imputation for nonresponse in surveys*1987: Wiley-IEEE.
